# A Countrywide Seroepidemiological Survey of Rift Valley Fever in Livestock, Uganda, 2017

**DOI:** 10.4269/ajtmh.22-0504

**Published:** 2023-07-31

**Authors:** Luke Nyakarahuka, Jackson Kyondo, Carson Telford, Amy Whitesell, Alex Tumusiime, Sophia Mulei, Jimmy Baluku, Caitlin M. Cossaboom, Deborah L. Cannon, Joel M. Montgomery, Julius J. Lutwama, Stuart T. Nichol, Stephen Balinandi, John D. Klena, Trevor R. Shoemaker

**Affiliations:** ^1^ Department of Arbovirology, Emerging and Reemerging Infectious Diseases, Uganda Virus Research Institute, Entebbe, Uganda;; ^2^ Department of Biosecurity, Ecosystems and Veterinary Public Health, Makerere University, Kampala, Uganda;; ^3^ Viral Special Pathogens Branch, Division of High-Consequence Pathogens and Pathology, US Centers for Disease Control and Prevention, Atlanta, Georgia

## Abstract

In 2016, an outbreak of Rift Valley fever was reported in the Kabale District in Uganda for the first time in 48 years. Three human cases were confirmed by polymerase chain reaction, and subsequent serological investigations revealed an overall IgG seropositivity of 13% in humans and 13% in animals. In response to this reemergence, we designed a countrywide survey to determine the seropositivity of anti-Rift Valley fever virus (RVFV) IgG antibodies in livestock. Samples were collected from 27 districts and tested for RVFV anti-IgG antibodies. A total of 3,181 livestock samples were tested, of which 54.4% were cattle (1,732 of 3,181), 34.3% were goats (1,091 of 3,181), and 11.3% were sheep (358 of 3,181). Overall RVFV seropositivity was 6.9% (221 of 3,181). Seroprevalence was greater in cattle (10.7%) compared with goats (2.6%) and sheep (2.0%), among females (7.5%) compared with males (5.2%), and among adults (7.6%) compared with juveniles (4.9%) and nurslings (6.4%). Exotic breeds and animals with a history of abortion or stillbirth also had greater odds of RVFV seropositivity. Animals grazed under tethering and paddocking had greater RVFV seropositivity compared with animals that grazed communally, and livestock in the western and eastern regions had the greatest seroprevalence. In a multivariate regression model, animal species (odds ratio [OR], 6.4; 95% CI, 3.5–11.4) and age (OR, 2.3; 95% CI, 1.4–3.6) were associated significantly with RVFV seropositivity. This study could be important in developing risk-based surveillance for early outbreak detection to limit the spread of RVFV in both human and animal populations.

## INTRODUCTION

Rift Valley fever (RVF) disease is caused by Rift Valley fever virus (RVFV), a single-stranded RNA virus in the order Bunyavirales, recently reclassified to the new family of Phenuiviridae and genus *Phlebovirus.* Rift Valley fever virus causes disease in humans and animals, and hence is an important pathogen of public and animal health, with economic consequences. It is transmitted mainly to animals by mosquitoes, especially *Aedes* and *Culex* species; however, other mosquito species have been described as vectors of RVFV.^1^ Human infection occurs most commonly through contact with infected body fluids of viremic animals.

Rift Valley fever virus infection can cause subclinical to severe disease in livestock, and severe outcomes are most frequent among immunologically naive populations. Such susceptibility is believed to be influenced by the type of animal production system, with livestock from free-range systems being less susceptible to severe outcomes relative to those from intensive production systems.^2^ Outbreaks among susceptible livestock populations result in large numbers of abortions and stillbirths, known as abortion storms. Abortion storms were reported in RVF outbreaks in Kenya during the 1990 s and have subsequently been linked as indicators of RVF outbreak spillover into human populations.^3^^,^^4^

Despite being near RVF-endemic countries of Tanzania and Kenya which have reported previous large epizootics and epidemics of RVFV, Uganda had not reported any laboratory-confirmed human cases of RVF since 1968. In 2016, after 48 years without a reported outbreak of RVF in Uganda, two human cases of RVF were confirmed in the abattoir workers in the southwestern district of Kabale.^5^ Subsequent outbreak investigations and regional serological studies indicated an anti-RVFV IgG seroprevalence of 13% in humans and 13% in animals in the Kabale District in 2016.^6^ Entomological investigations during the 2016 outbreak revealed polymerase chain reaction–positive *Culex* mosquitoes, indicating the presence of the virus in the Kabale ecosystem.^5^ After this outbreak investigation, we designed a countrywide serological survey to investigate RVFV seroprevalence among livestock (cattle, goats, and sheep) across Uganda, with an emphasis on border districts and those within the “cattle corridor,” which has a high population of domestic livestock. The specific objective of this study was to determine the seropositivity of RVFV in Ugandan livestock and to evaluate potential risk factors of past infection. This would help in targeting epidemic-prone areas for risk-based surveillance of RVFV to minimize spillovers into susceptible human and livestock populations.

## MATERIALS AND METHODS

**Figure 1.**
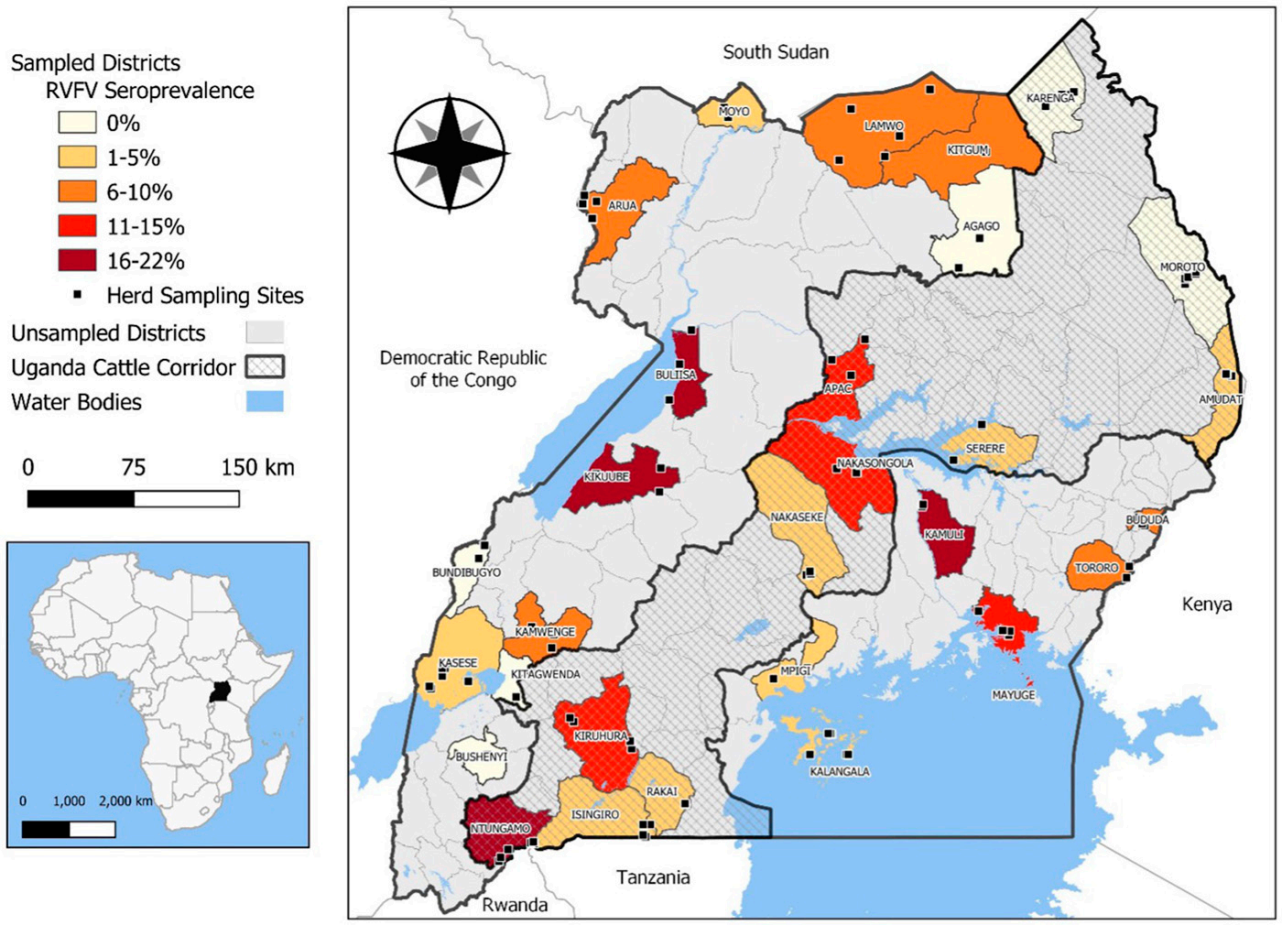
Map of Uganda showing sampled districts, seropositivity, cattle corridor of Uganda, and water bodies. RVFV = Rift Valley fever virus.

Cross-sectional sampling was conducted from March to August 2017 in 27 districts of Uganda. Blood samples were collected from livestock herds kept under sedentary husbandry that traveled relatively short distances for grazing. The selected districts were Rakai, Nakasongola, Kalangala islands, Nakaseke, and Mpigi in central Uganda; Serere, Mayuge, Bududa, Kamuli, and Tororo in Eastern Uganda; Apac, Arua, Moyo, Kaabong, Agago, Lamwo, Moroto, and Amudat, in northern Uganda; and Isingiro, Ntugamo, Bushenyi, Kamwenge, Kiruhura, Buliisa, Hoima, Bundibugyo, and Kasese in western Uganda. We selected a range of districts in Uganda to encompass various ecological contexts, including those within the cattle corridor and those bordering other countries. A map was created using QGIS 3.28.1 software to visualize the district-level seroprevalence and coordinates of herds that were sampled.^7^ Open-source shape files for Uganda district boundaries were downloaded from the Humanitarian Data Exchange (https://data.humdata.org/) (Figure 1).

### Sample size determination and sampling procedures.

We calculated the sample size according to our finding of 13% seroprevalence among livestock during outbreak investigations that occurred after the initial reemergence of RVFV in Uganda in 2016, with a margin of error of 3% and 95% confidence. It was necessary to include a design effect given the structured nature of sampling livestock within herds. We used a proportion-to-herd size sampling approach, sampling all animals in herds with ≤ 15 members, and only 25% of animals in herds with > 15 members. Assuming an average of 15 animals sampled per herd, and allowing for a high correlation of seropositivity among animals within the same herd using an intraclass correlation coefficient (ICC) of 0.3, we calculated 5.2 as the necessary design effect. Therefore, our calculated sample size was 2,511 livestock. We anticipated sampling ∼167 herds, with an average of 15 animals per herd, selected purposively based on greater risk factors for acquiring RVF, such as proximity to flooding areas, high levels of animal movement, and proximity to international borders.

Geographic coordinates were also recorded at each sampling site. Blood samples were collected in vacutainer tubes from either the jugular vein or the tail vein (middle caudal vein), and were aliquoted immediately and transferred into liquid nitrogen for cold storage. Samples were transported to the Uganda Virus Research Institute (UVRI) viral hemorrhagic fever laboratory for serological testing.

#### Laboratory testing.

The detection of anti-RVFV IgG antibodies was conducted by the UVRI VHF laboratory using a US CDC indirect ELISA method, as described in previous studies.^6^^,^^8^^,^^9^ Briefly, specimens were tested using anti-IgG ELISA using inactivated RVF-infected Vero E6 cells following heat inactivation. Four dilutions of each specimen were tested as follows: 1:100, 1:400, 1:1,600, and 1:6,400. Titers and cumulative sum optical densities (ODs) less the background observance of uninfected control antigens were recorded. Samples were deemed positive if the adjusted sum OD was > 0.95.

#### Data collection and analysis.

Animal independent variables, which included age, sex, breed, grazing pattern, location of the herd, herd size, and health history, were also collected using a data collection form. Data were entered into Excel (Microsoft Corporation, Redmond, WA) and analyzed using R version 4.0.2 (R Foundation for Statistical Computing, Vienna, Austria). Geographic coordinates were recorded at the location each herd was sampled and were used to extract elevation at each sampling location using data from WorldClim.^10^ The dependent variable of analysis was anti-RVFV IgG seropositivity as detected using ELISA. Associations between RVFV seropositivity and potential risk factors were measured in unadjusted bivariate analyses and an adjusted mixed-effects binomial generalized linear model, with a random effect for each herd sampled. We categorized elevation into low- or high-elevation agroecological zones using a cutoff of 1,200 m, which is a natural cutoff between vegetation land-use patterns that can affect disease vectors in Uganda.^11^ We selected reference groups for each variable as the strata with the lowest seroprevalence except for species, abortion, and stillbirth variables. Although sheep were the species with the lowest seroprevalence, their sample size was low; therefore, goats were used as the reference group. For the variables representing a history of stillbirth and abortion, the reference group included those that did not have a history. Variables with more than 5% missingness were excluded from the multivariate analysis. The country region was also excluded from the multivariate analysis because our goal was to identify animal characteristics associated with RVFV seropositivity. Formal spatial analyses of these data accounting for spatial correlation of herd-level seroprevalence are reported separately.^12^ The variance of the herd-level random effect was used to calculate the ICC to determine the extent to which animals within herds were similar with regard to RVFV seropositivity results. To calculate the ICC, we used

$$\text{ICC}=\frac{\sigma }{\left(\sigma +\pi^{2}\right)/3}\text{,}$$

where *σ* is the variance associated with each herd intercept. We followed the informed consent process with animal owners and herdsmen to obtain permission to sample their livestock.

## RESULTS

### Animal demographics.

Sampling enrollment was greater than expected, and we sampled 198 herds and 3,181 livestock, including 1,732 cattle (54.4%), 1,091 goats (34.3%), and 358 sheep (11.3%). Most animals were adults (70.6%), female (78.0%), and local/indigenous breeds (71.5%) (Table 1). Greater seropositivity was observed in districts neighboring water bodies, which are prone to flooding, such as around Lake Victoria and the Nile. In contrast, seropositivity was not significant in dry and mountainous areas, such as the Karamoja region in northeastern Uganda and the plains of Mount Rwenzori (Figure 1).

**Table 1:** Univariate analysis of animal demographics and overall seroprevalence

Variable	*n*	%
Species		
Cattle	1,732	54.5
Goats	1,091	34.3
Sheep	358	11.3
Age*		
Nurslings	376	11.8
Juvenile	556	17.5
Adult	2,247	70.6
Unknown	2	0.1
Sex		
Female	2,482	78.0
Male	688	21.6
Unknown	11	0.4
Breed		
Cross	836	26.3
Exotic	71	2.2
Local	2,274	71.5
RVF IgG result		
Negative	2,960	93.1
Positive	221	6.9
Current health status		
Healthy	2,685	84.4
Unhealthy	257	8.1
Unknown	239	7.5
Past health status		
Healthy	2,522	79.3
Unhealthy	109	3.4
Unknown	550	17.3
Grazing pattern		
Paddocking	842	26.5
Communal	1,430	45.0
Tethering	170	5.3
Zero grazing	113	3.6
Missing	626	19.7
Abortion		
No	683	21.5
Yes	696	21.9
Missing	1,802	56.7
Stillbirth		
No	985	31.0
Yes	341	10.7
Missing	1,855	58.3
Elevation		
High	1,403	44.1
Low	1,778	55.9
Region		
Eastern	526	16.5
Northern	923	29.0
Central	662	20.8
Western	1,070	33.6

RVF = Rift Valley fever.

* The age category was estimated qualitatively by looking at the physical attributes of sampled animals because rural farmers do not have records.

### RVF seropositivity.

The overall seropositivity among the three animal species was 6.9% (221 of 3,181). Unadjusted bivariate analysis of the individual relationships between RVF IgG seropositivity and each characteristic (Table 2) found that cattle had a greater seropositivity (10.7%) compared with goats (2.6%) and sheep (2.0%), and the odds of seropositivity among cattle was 4.57 times the odds among goats (95% CI, 3.1–7.0). Adult livestock had greater seropositivity compared with nurslings and juvenile animals; compared with the referent juvenile group, adults had significantly greater odds of seropositivity (odds ratio [OR], 1.2; 95% CI, 1.1–2.6). Females had significantly greater seropositivity (7.5%) compared with males (5.2%; OR, 1.5; 95% CI, 1.0–2.1). Compared with local breeds, which had a seroprevalence of 6.3%, exotic breeds had a significantly greater seropositivity of 16.9% (OR, 3.0; 95% CI, 1.5–5.5). Although there were no statistically significant differences in animal seropositivity and animal production systems, paddocking, tethering, and zero-grazing groups all had a greater seroprevalence compared with communal grazing. Animals that had a reported history of abortion were 1.8 times more likely to be seropositive compared with those that did not have a reported abortion (OR, 1.8; 95% CI, 1.3–2.7). However, animals that had a reported stillbirth were 50% less likely to be seropositive compared with those without a history of stillbirth. Animals sampled from areas of low elevation had a greater seroprevalence than those from high altitudes, although this difference was not statistically significant (OR, 1.2; 95% CI, 0.9–1.6). Animals sampled from the eastern and western regions of Uganda had a seroprevalence of 8.6% and 8.5%, respectively, both of which were significantly greater than the central region (Table 2).

**Table 2:** Bivariate analysis of RVFV

Variable	RVF negative		RVF positive		Unadjusted odds ratio (95% CI)	*P* value
	*n*	%	*n*	%		
Species						
Goats	1,063	97.4	28	2.6	Reference	Reference
Cattle	1,546	89.3	186	10.7	4.6 (3.1–7.0)	< 0.01
Sheep	351	98.0	7	2.0	0.8 (0.3–1.7)	0.51
Age						
Juvenile	530	95.3	26	4.7	Reference	Reference
Nurslings	352	93.6	24	6.4	1.4 (0.8–2.5)	0.26
Adult	2,076	92.4	171	7.6	1.2 (1.1–2.6)	0.02
Sex						
Male	652	94.8	36	5.2	Reference	Reference
Female	2,297	92.6	185	7.5	1.5 (1.0–2.1)	0.04
Breed						
Local	2,130	93.7	144	6.3	Reference	Reference
Cross	771	92.2	65	7.8	1.3 (0.9–1.7)	0.15
Exotic	59	83.1	12	16.9	3.0 (1.5–5.5)	< 0.01
Grazing pattern						
Communal	1,334	93.3	96	6.7	Reference	Reference
Paddocking	768	91.2	74	8.8	1.3 (1.0–1.8)	0.07
Tethering	152	89.4	18	10.6	1.7 (0.9–2.7)	0.07
Zero grazing	104	92.0	9	8.0	1.2 (0.6–2.3)	0.61
Abortion						
No	635	93.0	48	7.0	Reference	Reference
Yes	612	87.9	84	12.1	1.8 (1.3–2.7)	< 0.01
Stillbirth						
No	875	88.8	110	11.2	Reference	Reference
Yes	319	93.6	22	6.5	0.6 (0.3–0.9)	0.01
Elevation						
High	1,314	93.7	89	6.3	Reference	Reference
Low	1,646	92.6	132	7.4	1.2 (0.9–1.6)	0.23
Region						
Central	628	94.9	34	5.1	Reference	Reference
Northern	872	94.5	51	5.5	1.1 (0.7–1.7)	0.73
Eastern	481	91.4	45	8.6	1.7 (1.1–2.8)	0.02
Western	979	91.5	91	8.5	1.7 (1.2–2.6)	< 0.01

RVF = Rift Valley fever; RVFV = Rift Valley fever virus.

Variables that were included in the adjusted, multivariate binomial generalized linear mixed regression model included animal species, age, sex, breed, and elevation (Table 3), whereas grazing pattern, abortion history, stillbirth history, current health status, and past health status were excluded because of > 5% missing data. After adjusting for the effect of all other variables included in the model, we found that animal species remained significantly associated with RVFV seropositivity; the odds of RVFV seropositivity among cattle were 6.3 times the odds among the goat reference group (95% CI, 3.5–11.4). Animal age was also associated significantly with seropositivity, where the odds of seropositivity among adult livestock were 2.3 times the odds among juveniles (95% CI, 1.4–3.9). In contrast to the unadjusted bivariate analysis, no statistically significant difference between male and female animals was found. Similarly, no animal breeds were found to have significantly greater odds of RVFV seropositivity compared with local breeds in the multivariate analysis. We found a very high correlation in seropositivity results among animals within herds, with an ICC of 0.42.

**Table 3:** Multivariate logistic regression analysis of RVFV seropositivity

Variable	Odds ratio (95% CI)	*P* value
Species		
Goats	Reference	Reference
Cattle	6.3 (3.5–11.4)	< 0.01
Sheep	1.2 (0.5–3.1)	0.73
Age		
Juvenile	Reference	Reference
Nurslings	0.7 (0.4–1.4)	0.31
Adult	2.3 (1.4–3.6)	< 0.01
Sex		
Male	Reference	Reference
Female	1.2 (0.7–1.8)	0.54
Breed		
Local	Reference	Reference
Cross	0.8 (0.4–1.5)	0.52
Exotic	2.2 (0.7–7.1)	0.20
Elevation		
High	Reference	Reference
Low	1.4 (0.6–2.8)	0.43

RVFV = Rift Valley fever virus.

## DISCUSSION

We report the results of the largest nationwide seroprevalence survey of RVFV in livestock in Uganda—a country that, before 2016, had not reported RVFV in humans or animals for almost 50 years. We sampled and tested 3,181 livestock samples using the CDC-developed anti-RVFV IgG ELISA. Overall seroprevalence in the three sampled livestock types was 6.9%, with district-level seroprevalence ranging from 0% to 22%. Previous studies have reported an overall seroprevalence of 10% to 16% in Uganda.^3^^,^^13^ This variation in seropositivity presented by different studies could be related to the use of commercial assays and the calibration, interpretation, and varying protocols used to detect IgG RVFV antibodies in animals. Alternatively, different sampling strategies could also cause these differences, as some studies are based on outbreak areas and sampling in high-risk areas. Given our finding of a very high correlation of past exposure status within herds, we would expect such variability depending on the number of herds sampled. We sampled from both low- and high-prevalence areas, and exceeded our goals for sample size and the number of herds sampled throughout the country, finding substantial heterogeneity between herds and across districts.

Although the overall seropositivity was ∼7%, greater seropositivity was observed in cattle (10.7%), and lower seropositivity was seen in goats (2.7%) and sheep (1.9%). This is in agreement with other studies that detected a lower seroprevalence of RVF in small ruminants (goats and sheep) compared with cattle.^13^ However, a study in central Uganda found a greater seroprevalence (9.8%) in goats.^14^ The greater seroprevalence in cattle in our study could be because cattle live longer and tend to be grazed over larger distances from their primary farm or home location. In contrast, goats and sheep tend to be paddocked closer to their primary homesteads and not grazed far from these locations. A greater infection rate in cattle could have negative economic impacts, because cattle are of the greatest market value to producers compared with small ruminants. Rift Valley fever is a transboundary disease with international trade concerns, leading to restrictions usually imposed on countries that are RVF endemic. This is of concern to many governments, especially when it comes to the international trade of animals and animal products. To reduce the economic burden, measures to prevent and control RVF transmission could be targeted toward these susceptible species with high seropositivity. To date, no livestock vaccination programs for RVF have been initiated in Uganda, in part because of the lack of a readily available and effective vaccine.^15^^–^^18^

Rift Valley fever IgG seropositivity was greater in female animals compared with male animals, which could be because female animals tend to live longer in herds for reproduction purposes as opposed to males, leading to greater rates of exposure and probability of being infected during their lifetime. Exotic (nonindigenous) breeds of animals had a greater prevalence, at 16%, compared with the local or crossbreeds in this study (*P* = 0.01). This could be a result of the exotic breeds being immunologically naive and having low resistance, as has been shown in other infections such as Nagana disease. However, our sample size of exotic-breed animals was low, and additional data should be collected to determine whether exotic breeds truly have greater odds of RVFV infection.^19^ It was evident that the history of abortion was associated significantly with RVFV IgG seropositivity (OR, 1.8; 95% CI, 1.3–1.7), as RVFV infection is known to cause reproductive complications, including abortions, in livestock.^20^ Investigating the causes of abortions in livestock is challenging, given that various infectious agents are endemic in Uganda that can result in such outcomes, including brucellosis or trichomoniasis. Future surveillance efforts are planned to prioritize the identification of suspect causes of abortions in animals to be able to understand the role of RVFV in causing livestock abortions in the RVFV-endemic regions of Uganda.

Regarding the spatial distribution and environmental drivers of RVF among livestock in Uganda, we found that seroprevalence was greatest in the western region compared with the central, eastern, and northern regions, and the odds of RVF seroprevalence were significantly greater in livestock from the western region of Uganda compared with central Uganda in the unadjusted bivariate analysis (OR, 2.5; *P* < 0.01). Rift Valley fever seroprevalence was also greater in areas at lower elevations (7.5%) compared with higher elevation areas (6.3%). Low-elevation areas would favor the accumulation of water as a result of rain runoff and flooding, which is conducive for vector survival and transmission to animals, especially during years with increased precipitation, such as during El Niño years.^21^^,^^22^ Notably, districts in the western region of Uganda had greater seropositivity, as did areas along the Nile and near large water bodies and wetland regions at low altitudes, which are susceptible to flooding (Figure 1). Dry areas in the northeastern regions of Karamoja and the western mountain areas of Rwenzori had a lower seroprevalence. This would suggest competent mosquito vectors and/or their larvae do not survive well in these high-altitude areas and transmission is limited (*P* = 0.01).

Because RVF infection can cause severe disease in humans, it would be beneficial to heighten surveillance in the animal populations to identify outbreaks before viral spillover into human populations. Regions and livestock groups highlighted in our study as having high positivity could be targeted for enhanced surveillance to limit the transmission and further spread of the disease. Our study provides evidence that forms a framework that can be used for high-risk–based surveillance of RVFV. Data obtained from this study could also be used for predictive risk mapping to create decision support tools for use in policy, planning, and disease control relevant to human and animal health agencies in Uganda.

Our study is limited in interpretation because sampling was purposive and biased toward high-risk and low-risk areas. There is a need for future sampling in areas that have low livestock populations and minimal animal movement dynamics. There is also a need to develop field-based diagnostics that can be used by district veterinary officers to help with the early detection of sick herds, and that can be used for investigations during outbreaks of RVF. An animal management plan that incorporates laboratory detection, animal husbandry, and control measures should be a priority for countries at risk for RVF infections. In addition, several animal-level characteristics had high proportions of missing data and were not able to be included in the adjusted multivariate analysis of potential risk factors associated with RVFV seropositivity. Due to missing data, the multivariate model results were not adjusted for potentially significant predictors of RVFV seropositivity, such as a history of abortion and stillbirth.
